# A Case of Neonatal Neutropenia Due to Anti-Fc Gamma Receptor IIIb Isoantibodies Treated with Recombinant Human Granulocyte Colony Stimulating Factor

**DOI:** 10.1155/2009/717545

**Published:** 2009-08-26

**Authors:** Maja Tomicic, Mirta Starcevic, Vanja Zach, Jasna Bingulac-Popovic, Zeljka Hundric-Haspl

**Affiliations:** ^1^Department of Platelet and Leukocyte Immunology, Croatian Institute of Transfusion Medicine, HR-10000 Zagreb, Croatia; ^2^Department of Neonatology, University Department of Pediatrics, Sestre Milosrdnice University Hospital, HR-10000 Zagreb, Croatia; ^3^Department of Molecular Immunogenetics, Croatian Institute of Transfusion Medicine, HR 10000, Zagreb, Croatia; ^4^Department of Immunohematology, Croatian Institute of Transfusion Medicine, HR 10000, Zagreb, Croatia

## Abstract

Alloimmunization to granulocyte-specific antigens can occur during pregnancy. Maternal antibodies of IgG class can cross the placenta to result in alloimmune neonatal neutropenia. Antibodies to human neutrophil antigens anti-HNA-1a, HNA-1b, and HNA-2a have been most commonly reported to cause alloimmune neonatal neutropenia. Isoantibodies to Fc gamma RIIIb (CD16) if mother is a HNA-null phenotype are rarely involved in neonatal neutropenia. We report on a case of severe neutropenia (440 neutrophils/*μ*L) due to anti-Fc gamma RIIIb (CD16) isoimmunization. On day 14 severe omphalitis developed, which was treated for 7 days by an antibiotic (ceftriaxone in a dose of 80 mg/kg/d) according to umbilical swab finding. Omphalitis persisted for 10 days in spite of antibiotic therapy and only resolved upon the introduction of rhG-CSF therapy. Therapy with rh-GCSF proved efficient and led to neutrophil count increase to 1970/*μ*L and cure of omphalitis. However, therapeutic effect on granulocyte count was of transient nature, as granulocyte count fell to 760 n/*μ*L on day 4 of therapy discontinuation. Neutropenia persisted for 2 months. The newborn was discharged from the hospital on day 26 with normal clinical status with clinical and laboratory control examinations at 2-week intervals. No additional infections were observed during the course of neutropenia.

## 1. Introduction

Alloimmune neonatal neutropenia (ANN) is an uncommon but potentially life-threatening disorder of the neonate [1]. The incidence of ANN has been estimated to 1 *per* 1000–6000 live births [2]. ANN is the result of maternal alloimmunization to granulocyte antigens. The passive transfer of maternal neutrophil-specific antibodies and subsequent sensitization of fetal neutrophils can result in severe neutropenia of the neonate [2].

Antibodies to granulocyte-specific antigens HNA-1a and HNA-1b have been most commonly reported to cause ANN. Anti-HNA-2a and antibodies to Fc gamma RIIIb (CD16), if mother is a HNA-1-null phenotype are rarely involved in neonatal neutropenia [3–5].

ANN should be suspected in a newborn with isolated neutropenia (<1500 cells/*μ*L) [4, 5].

The course of pregnancy is uneventful, and mother has normal granulocyte count and no clinical history of frequent bacterial infections [2].

The clinical course is usually self-limiting with only mild infection; however, in severe cases complicated with bacterial sepsis it is a potentially life-threatening disorder [3].

Demonstration of alloantibodies against granulocyte-specific antigen shared by neonatal and paternal granulocytes in maternal serum is essential in the diagnosis of ANN. Human neutrophil antigen (HNA) genotyping of mother and father can be useful in supporting serology results [6–11]. 

The treatment usually includes antibiotics, intravenous gamma globulins, and in severe cases of ANN complicated with bacterial sepsis therapy with rhG-CSF is indicated, however, with a variable success [12–15].

The issue of the choice and efficacy of specific therapy to increase the blood neutrophil count in the management of ANN is not fully defined. 

## 2. Case Report

A male newborn, birth weight 3540 g, was born from second, uncomplicated pregnancy, to a healthy 29-year-old mother, in 40th week of gestation. Mother had a normal neutrophil count. First borne child was healthy. Mother’s sister is of the same HNA-1-null genotype, delivered two healthy children. 

Severe neutropenia (440 neutrophils/*μ*L) with normal findings of other laboratory tests was detected on the first day of the newborn's life.

On day 14 severe omphalitis developed, which was treated for 7 days by an antibiotic (ceftriaxone in a dose of 80 mg/kg/d) according to umbilical swab finding. 

**Figure 1:**
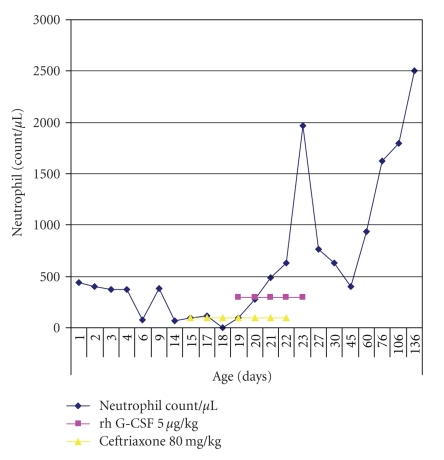
The neonate's neutrophil count pattern and hr-G-CSF therapy course.

Upon making the diagnosis of neonatal isoimmune neutropenia due to anti-Fc gamma RIIIIb (CD16) pan-reactive antibodies, therapy with rh-GCSF from day 19 to day 23 (Neupogen in a dose of 5 *μ*g/kg/d) was introduced (Figure 1). Therapy with rh-GCSF proved efficient and led to neutrophil count increase to 1970/*μ*L and cure of omphalitis. However, therapeutic effect on granulocyte count was of transient nature, as granulocyte count fell to 760 neutrophils/*μ*L on day 4 of therapy discontinuation. Neutropenia persisted for 2 months (Figure 1). The newborn was discharged from the hospital on day 26 with normal clinical status with clinical and laboratory control examinations at 2-week intervals. No additional infections were observed during the course of neutropenia.

## 3. Serology

Serologic studies of the mother's and newborn's sera included granulocyte immunofluorescence tests, direct and indirect (GIFT-DT, IT), granulocyte agglutination test (GAT), and monoclonal antibody immobilization of granulocyte antigens (MAIGAs) for granulocyte antibody screening and identification [6–11].

IgG class antigranulocyte antibodies were detected by GIFT in the maternal and neonatal sera. Results of serologic testing are summarized in Table 1 showing anti-CD 16 pan-reactive antibodies in the maternal and newborn’s sera.

Antibody identification is definitely verified by determination of maternal, paternal, and neonatal HNA by polymerase chain reaction-sequence-specific primers (PCR-SSP) [10, 11]. Following primers were used:

HNA-1A 5′-CAG TGG TTT CAC AAT GTG AA -3′
HNA-1AR 5-ATG GAC TTC TAG CTG CAC -3′
HNA-1-B 5′-CAA TGG TAC AGC GTG CTT -3′
HNA-1BR 5′-ACT GTC GTT GAC TGT GTC AG- 3′
HNA-1C 5′- AAG ATC TCC CAA AGG CTG TG - 3′
HNA-1CR 5′- ACT GTC GTT GAC TGT GTC AT -3′
HGH-1 5′-CAG TGC CTT CCC AAC CAT TCC CTT A -3′
HGH-2 5′-ATC CAC TCA CGG ATT TCT GTT GTG TTT C -3′

The presence of 439-bp fragment of the human growth hormone gene indicated that amplification took place properly, and no nonspecific reactions were observed.

**Figure 2:**
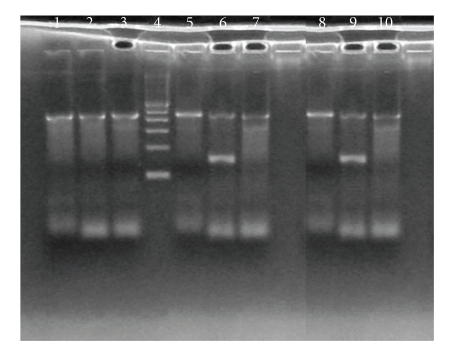
HNA-1 genotyping by PCR-SSP method, Electrophoresis of HNA-1 allele PCR products on 1.5% agarose gel for three subjects and three HNA alleles 1a, 1b, and 1c. Mother: lanes 1, 2, and 3, genotype HNA-1a negative, 1b negative, 1c negative; lane 4 molecular marker 100 bp; Father: lanes 5, 6, and 7, genotype is HNA-1a negative, 1b positive, 1c negative; Newborn: lanes 8, 9, and 10, genotype HNA-1a negative, 1b positive, 1c negative; human growth hormone (HGH) gene fragment of 439 bp used as internal positive control; the amplified HNA-1b allele positive product 155 bp.

HNA genotyping of the mother showed Fc gamma RIIIb deficiency (HNA-1a=, 1b=, 1c=). The newborn and father were genotyped as HNA-1a=, 1b+, 1c= confirming the diagnosis of isoimmune neonatal neutropenia. Results are shown on Figure 2.

## 4. Discussion

We report on a case of severe neonatal neutropenia due to anti-Fc gamma RIIIb (CD16) isommunization in the child born from the second uneventful pregnancy to healthy mother. 

Several cases of immune neonatal neutropenia caused by anti-Fc gamma RIIIb isoantibodies have been described in literature [4, 16–19]. Fromont et al. report on the presence of iso-anti-CD-16 antibodies in one of five individuals with Fc gamma RIIIb deficiency (three of them pregnant women), all of them being healthy [17]. Haas et al. analyzed history data of 21 donors with Fc gamma RIIIb deficiency identified in 14 unrelated families. Recurrent bacterial infections were recorded in 3/21 subjects, whereas others had never had any serious bacterial infection [4]. 

In the case presented, a severe form of omphalitis was observed on postnatal day 14 and persisted for 10 days of antibiotic therapy introduction, as indicated by the antibiotic sensitivity report followed by rhG-CSF therapy. Omphalitis persisted in spite of antibiotic therapy and only resolved upon the introduction of rhG-CSF therapy. 

The issue of the choice and efficacy of specific therapy to increase the blood neutrophil count in the management of ANN is not fully defined [20]. The effect of prophylactic antibiotic therapy, intravenous immunoglobulin, and recombinant human granulocyte colony-stimulating factor (rh-GCSF) is variable and may prove useful in some cases [20, 21].

According to literature data, clinical experience with rh-GCSF use is highly favourable but mostly referring to the management of neutropenia following bone marrow and stem cell transplantation neonatal sepsis and autoimmune neutropenia [22, 23]. Experience with rh-GCSF in the treatment of ANN is quite limited, and the effect of rh-GCSF on developing tissues of the neonate is unknown [24, 25]. In the majority of ANN cases, the use of rh-GCSF resulted in a very rapid and steady neutrophil count increase [26].

In our patient, therapy with rh-GCSF proved efficient and led to an increase in neutrophil count and resolution of omphalitis. However, therapeutic effect on granulocyte count was transient, since granulocyte count showed a decline on day 4 of therapy discontinuation. 

The child was discharged for home care with clinical and laboratory control examinations at 2-week intervals. No further bacterial infections were observed during the 8-week period of neutropenia. Therefore, we decided to follow up the course of the disease and to reintroduce rh-GCSF therapy in case of severe bacterial infection or sepsis development.

We believe that in case of a mild clinical course of the disease, a neonate benefits more from being discharged for home care than from insisting on normal neutrophil count achievement associated with prolonged hospital stay and potential exposure to hospital infections. Yet, we are fully aware of the risk of severe and protracted neutropenia. This case report may hopefully present a step forward to elucidate the issue.

## Figures and Tables

**Table 1:** Results of serologic testing.

	GIFT IT	GIFT DT	GAT IT	MAIGA CD16 Fc gamma RIIIb	MAIGA CD177	MAIGA CD11 a/11 b	MAIGA Beta-2microglobulin
Maternal							
Serum	pos		pos	pos, anti-HNA-1 pan-reactive	neg	neg	pos, anti-HLA-class I
Granulocytes		neg					

Newborn							
Serum	pos		pos	pos, anti-HNA-1 pan-reactive	neg	neg	pos, anti-HLA-class I
Granulocytes		pos					

GIFT = granulocyte immunofluorescence test, GAT = granulocyte agglutination test, MAIGA = monoclonal antibody immobilization of granulocyte antigens, IT = indirect test, DT = direct test, Fc gamma RIIIb = Receptor Fc gamma IIIb, pos = positive, neg = negative, HNA = human neutrophil antigen, and HLA = human leukocyte antigen.
